# Exploring the Determinants of Mobile Health Adoption by Hospitals in China: Empirical Study

**DOI:** 10.2196/14795

**Published:** 2020-07-14

**Authors:** Boumediene Ramdani, Binheng Duan, Ilhem Berrou

**Affiliations:** 1 Centre for Entrepreneurship College of Business & Economics Qatar University Doha Qatar; 2 Creative Assembly Spire Court, Albion Way Horsham United Kingdom; 3 Faculty of Health and Applied Sciences University of the West of England Bristol United Kingdom

**Keywords:** mHealth, mobile phone, adoption, hospitals, TOE, China

## Abstract

**Background:**

Although mobile health (mHealth) has the potential to transform health care by delivering better outcomes at a much lower cost than traditional health care services, little is known about mHealth adoption by hospitals.

**Objective:**

This study aims to explore the determinants of mHealth adoption by hospitals using the technology-organization-environment (TOE) framework.

**Methods:**

We conducted an interviewer-administered survey with 87 managers in Chinese public hospitals and analyzed the data using logistic regression.

**Results:**

The results of our survey indicate that perceived ease of use (β=.692; *P*<.002), system security (β=.473; *P*<.05), top management support (β=1.466; *P*<.002), hospital size (β=1.069; *P*<.004), and external pressure (β=.703; *P*<.005) are significantly related to hospitals’ adoption of mHealth. However, information technology infrastructure (β=.574; *P*<.02), system reliability (β=−1.291; *P*<.01), and government policy (β=2.010; *P*<.04) are significant but negatively related to hospitals’ adoption of mHealth.

**Conclusions:**

We found that TOE model works in the context of mHealth adoption by hospitals. In addition to technological predictors, organizational and environmental predictors are critical for explaining mHealth adoption by Chinese hospitals.

## Introduction

### Background

The aging population and the high prevalence of complex long-term conditions are placing unprecedented pressure on hospital services in China [1,2]. Mobile health (mHealth) not only has the potential to alleviate pressure on hospital services but can also increase accessibility and meet individual patient demands. It has been advocated as a complementary approach to traditional (ie, offline) health care services [3]. With over 1.3 billion mobile subscribers [4], mHealth services in China are considered the largest market in the world, accounting for 12.53 billion yuan or US $1.76 billion (1 yuan = US $0.14), in 2017 [5]. Defined as “the use of mobile devices—such as mobile phones, patient monitoring devices, personal digital assistants (PDAs) and wireless devices—for medical and public health practice” [6], mHealth has the potential to transform health care by delivering better outcomes at a lower cost [7]. For patients, mHealth has the potential to improve the health and well-being of individuals by recognizing behaviors, providing a rapid diagnosis of medical conditions, delivering just-in-time interventions, and continuous monitoring of their health status [8]. Recent evidence shows that mHealth could improve patient experience [9]. For health care providers, mHealth could reduce demands on clinicians’ time by minimizing office visits for the management of common conditions and enabling patient self-management [7].

China is a particularly interesting context for this study. In 2015, 1 in 4 persons aged ≥60 years lived in China, making it the largest population of older citizens in the world [10]. This trend is projected to grow by 71% between 2015 and 2030. Moreover, medical institutions in China are concentrated in cities, making it difficult to deliver health care services to the rural population [11]. Chinese policymakers need to address these challenges and find an effective solution that reaches both the elderly and rural populations. For that, mHealth is part of a national strategy to resolve the “difficulty and expense of seeking a doctor” [12].

Chinese hospitals have begun to take up mHealth to deliver health care services [3,9,12,13]. Most emerging literature focuses on patients’ adoption of mHealth services. However, little is known about the hospitals’ adoption of mHealth. Therefore, this study aims to address this gap by exploring the determinants of mHealth adoption by hospitals.

A large body of research has explored the determinants of adopting health care technologies. As highlighted in Table 1, previous research explored various health care technologies using several theoretical lenses in different settings. From reviewing the literature, it is still unclear what determines the adoption of mHealth by hospitals.

**Table 1:** Adoption of health care technologies.

Adoption theory	Adoption of technology^a^	Constructs/factors^b^	Method	Data	Location [reference]
TAM^c^ with trust and perceived risks	mHealth^d^	Perceived usefulness, perceived ease of use, perceived risk, performance risk, legal concerns, and trust	SEM^e^	388 patients in large hospitals	China [12]
TAM and UTAUT^f^	Medical dashboard system	Performance expectancy, effort expectancy, social influence, and facilitating conditions	SEM	383 physicians and nurses in a tertiary teaching hospital	Korea [14]
TAM and UTAUT	Mobile electronic medical record	Performance expectancy, effort expectancy, attitude, social influence, facilitating conditions, and behavior intention to use	SEM	449 subjects (65 physicians and 385 nurses) in a large tertiary hospital	Korea [15]
UTAUT 2	mHealth	Performance expectancy, effort expectancy, social influence, facilitating conditions, hedonic motivation, price value, habit, waiting time, and self-concept	Factor analysis and path analysis	A total of 3 surveys with 387, 359, and 375 patients who were offered mHealth as an alternative to traditional hospital services	United States, Canada, and Bangladesh [16]
Task-technology fit and social contagion theory	EHR^g^	Authorization, compatibility, data quality, ease of use, information systems relationship, timeliness, locatability, system reliability, and social contagion	SEM	Survey with 51 university students with working experience in the health care sector and used EHR systems in the past	United States [17]
UTAUT	Home health care robots	Performance expectancy, effort expectancy, social influence, facilitating conditions, trust, privacy concerns, ethical concerns, and legal concerns	SEM and power analysis	108 health care professionals and patients working for home health care agencies	United States [18]
Social capital theory, social cognitive theory and TAM	Telehealth	Perceived ease of use, perceived usefulness, system self-efficacy, social participation, institutional trust, and social trust	SEM	365 patients who used a telehealth system for at least one month	Taiwan [19]
TPB^h^	mHealth service	Perceived value, attitude, perceived behavior control, subjective norm, perceived physical condition, resistance to change, technology anxiety, and self-actualization need	SEM	424 middle-aged and older people accessing community service centers	China [20]
UTAUT	HIT^i^	Performance expectancy, effort expectancy, social influence, facilitating conditions, and provincial areas	SEM	400 health care professionals working in hospital	Thailand [21]
No specific theory	mHealth usage intention, assimilation, and channel preferences	Individual difference, health care availability and health care utilization, and socioeconomic status and demographics	Hierarchical ordinary least squares	1132 consumers	United States [22]
Decomposed TPB and value-attitude-behavior	MEDLINE system	Perceived usefulness, perceived ease of use, attitude, interpersonal influence, subjective norm, personal innovativeness in IT^j^, self-efficacy, facilitating conditions, perceived behavioral control, and usage intention	SEM	224 physicians in primary care centers and hospitals	Taiwan [23]
TAM and TPB	Mobile health care	Attitude, perceived behavioral control, subjective norm, perceived usefulness, perceived ease of use, personal innovativeness, and perceived service availability	SEM	140 health care professionals working in hospitals	Taiwan [24]
UTAUT	HIT	Performance expectancy, effort expectancy, social influence, voluntariness, facilitating conditions, experience, and IT knowledge	SEM	Information management officers or head officers from 1323 community health centers	Thailand [25]
TAM and innovation diffusion theory	Electronic logistics information system	Compatibility, perceived usefulness, perceived ease of use, trust, perceived financial cost, and behavioral intention	SEM	Nurses working in 10 hospitals who used electronic logistics information system	Taiwan [26]
TAM	Telemedicine	Perceived usefulness and perceived ease of use	SEM	408 physicians working in tertiary hospitals	Hong Kong [27]

^a^Dependent variable.

^b^Independent variables.

^c^TAM: technology acceptance model.

^d^mHealth: mobile health.

^e^SEM: structural equation modelling.

^f^UTAUT: unified theory of acceptance and use of technology.

^g^EHR: electronic health record.

^h^TPB: theory of planned behavior.

^i^HIT: health information technology.

^j^IT: information technology.

### Research Model and Hypotheses

The technology-organization-environment (TOE) framework, first introduced by Tornatzky and Fleischer [28], has been used as a guiding theoretical basis for the adoption of many technologies. This framework integrates the 3 vital contexts—technology, organization, and environment—to provide a holistic understanding of adoption of technology from an organizational perspective. The generic nature of the framework allows researchers to explore the determinants that are relevant to their specific context. Moreover, it has the empirical support of several studies exploring the adoption of technology in different types of organizations and different sectors of the economy (Table 2). A number of technologies have been examined from open systems and electronic business (e-business) to enterprise systems, radio-frequency identification (RFID), and cloud computing. Most of these studies focused on the adoption of technology from an organizational perspective, including government agencies [29], large firms [30-32], and small-to-medium–sized enterprises (SMEs) [33-37]. Other studies focused on particular sectors, including hotels [38], retailing [39], manufacturing [38,40], and the services sector [40]. Surprisingly, only two studies addressed the adoption of technology from a hospital perspective [41,42]. This study aims to extend this body of research by using the TOE model to explore the determinants of mHealth adoption by hospitals.

**Figure 1:**
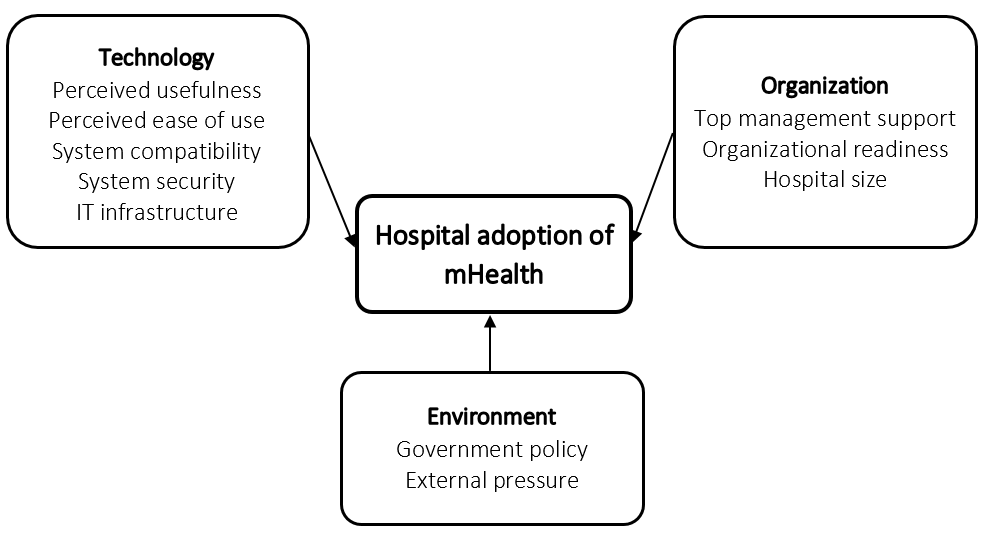
Technological organizational and environmental determinants of mHealth adoption by hospitals.

From reviewing the literature, we developed a TOE framework to explore mHealth adoption by hospitals in China. Our framework (Figure 1) suggests that hospital adoption of mHealth is influenced by TOE determinants: technology—perceived usefulness, perceived ease of use, system compatibility, system security, and information technology (IT) infrastructure; organization—top management support (TMS), organizational readiness, and size; and environment—government policy and regulations, and external pressure. Each of the 3 contexts is discussed below to develop our hypotheses.

**Table 2:** Technology-organization-environment determinants of adoption of technology.

Adoption of technology^a^ [reference]	Determinants^b^											
	Technology					Organization				Environment		
	Perceived usefulness	Perceived ease of use	Compatibility	System security	IT^c^ infrastructure		Top management support	Organizational readiness	Size		Government policy	External pressure
Cloud computing [29]	N/A^d^	N/A	N/A	N/A	X^e^		X	N/A	N/A		X	X
e-SCM^f^ [30]	X	N/A	N/A	N/A	N/A		X	X	N/A		N/A	X
Open systems [32]	N/A	N/A	N/A	N/A	X		N/A	N/A	N/A		N/A	X
Electronic data interchange [33]	X	N/A	N/A	N/A	X		N/A	N/A	N/A		X	X
Internet [34]	X	N/A	N/A	N/A	X		N/A	N/A	N/A		N/A	N/A
Enterprise systems [35]	X	N/A	N/A	N/A	N/A		X	X	X		N/A	N/A
Enterprise applications [36]	X	X	X	N/A	N/A		X	X	X		N/A	X
Enterprise resource planning [37]	X	N/A	X	X	X		N/A	N/A	X		X	X
Mobile reservation systems [38]	X	X	X	N/A	X		X	N/A	X		N/A	X
e-Business^g^ [39]	N/A	N/A	N/A	N/A	X		N/A	N/A	X		X	X
Cloud computing [40]	X	X	N/A	N/A	N/A		X	N/A	X		N/A	N/A
Health information exchange [41]	N/A	N/A	N/A	N/A	X		N/A	N/A	N/A		N/A	X
RFID^h^ [42]	X	N/A	X	X	N/A		X	X	N/A		X	X
Cloud computing [43]	N/A	X	N/A	N/A	X		N/A	N/A	N/A		N/A	X
RFID [44]	X	N/A	N/A	N/A	N/A		X	N/A	X		N/A	N/A
RFID [45]	X	X	X	N/A	X		X	N/A	X		N/A	X
e-Business [46]	X	N/A	X	X	X		N/A	N/A	X		N/A	X
e-Business [47]	N/A	N/A	N/A	N/A	X		X	N/A	X		X	X
e-Commerce^i^ [48]	X	N/A	N/A	N/A	N/A		N/A	N/A	N/A		X	X
e-Commerce [49]	N/A	N/A	N/A	N/A	X		N/A	N/A	N/A		X	X

^a^Dependent variable.

^b^Independent variables.

^c^IT: information technology.

^d^N/A: not applicable.

^e^X: significant determinant.

^f^e-SCM: electronic supply chain management.

^g^e-Business: electronic business.

^h^RFID: radio-frequency identification.

^i^e-Commerce: electronic commerce.

#### Technology

A number of technological determinants have been shown to affect organizational adoption of new technologies, including perceived usefulness, perceived ease of use, system compatibility, system security, and IT infrastructure. New technology is more likely to be adopted when an organization perceives it to offer more benefits than existing systems. Several new technologies have proven their advantage over existing practices, including e-business over physical stores [31,46], RFID over manual data entry [44,45], cloud computing over client-server computing [40], and mobile reservation over the telephone or web-based reservations [38]. In the health care context, the perceived benefits of RFID in keeping track of hospital patients were found to be a significant determinant of adoption [42]. In their study of customer relationship management (CRM), Hung et al [23] also found a relative advantage to be positively associated with CRM adoption by hospitals. On this basis, we suggested the following:

Hypothesis 1: Perceived usefulness will positively influence hospitals’ adoption of mHealth.

Perceived ease of use has been found to be a significant factor in the adoption of technology [36,38,40,43,45]. The widespread use of smartphones and health monitoring devices has made it easier for consumers to handle such devices remotely [16]. For hospitals to have a more active engagement in health care delivery, we expect the perceived ease of use of mobile technologies to be positively associated with mHealth adoption. Thus, we proposed the following:

Hypothesis 2: Perceived ease of use will positively influence hospitals’ adoption of mHealth.

System compatibility has been found as a significant determinant for the adoption of technology [36-38,45,46]. In the health care context, compatibility with a non-RFID–based patient tracking system was found to be a crucial determinant for RFID application integration [42]. Therefore, we suggested the following hypothesis:

Hypothesis 3: System compatibility with existing systems will positively influence hospitals’ adoption of mHealth.

Unauthorized data access is a concern for organizations and their clients and has the potential to jeopardize information security and privacy [50]. Similar to e-business, mobile technologies are integrated into transactions that involve fund transfer and the exchange of organizational data [46]. Although a few studies [37,46] found system security to be positively associated with the adoption of technology, it is of particular significance for health care providers. Security and privacy protection were found to be major determinants of RFID adoption by hospitals [42].A security breach of patient information not only puts patients at risk but can also cause a lasting damage to a hospital’s reputation. Thus, we proposed the following:

Hypothesis 4: System security will positively influence hospitals’ adoption of mHealth.

Finally, IT infrastructure has been found to be one of the most significant determinants of the adoption of technology. This factor was found to be significant for most types of technologies, including open systems [32], e-business [31,46], RFID [45], enterprise resource planning [37], mobile reservation systems [38], and cloud computing [29]. In a study of health information exchange (HIE), hospitals that do not have the necessary technological infrastructure were found to be less likely to adopt new systems [41]. Therefore, we expect IT infrastructure to be a significant determinant of mHealth adoption by hospitals, and we proposed the following hypothesis:

Hypothesis 5: IT infrastructure will positively influence hospitals’ adoption of mHealth.

#### Organization

TMS, organizational readiness, and organizational size have been found to influence the organizational adoption of new technologies. TMS has been found to be one of the most significant determinants of the adoption of technology [51] as managers can overcome barriers to adoption and resistance to change [30]. It has been found to be an influential factor in the adoption of e-business [46], enterprise systems [35], RFID [44,45], and mobile reservation systems [38]. In hospitals, TMS has been shown to be a significant determinant of RFID adoption for patient tracking [42]. Thus, we suggested the following:

Hypothesis 6: TMS will positively influence hospitals’ adoption of mHealth.

Organizational readiness, the degree to which an organization has the knowledge and resources that can remove barriers to system adoption [52], has been shown to be positively related to the adoption of new technology [30]. It has been found to influence the adoption of enterprise systems significantly [35,36]. In the health care context, organizational readiness has been shown to be of critical importance in RFID adoption [42]. Therefore, we proposed the following:

Hypothesis 7: Organizational readiness positively influences hospitals’ adoption of mHealth.

Organizational size is another important determinant of adoption of technology [51]. It was established that larger firms are more likely to adopt enterprise systems [35-37]. Other studies also confirm the significance of organizational size [38-40,45-47]. Thus, we suggested the following:

Hypothesis 8: Hospital size will positively influence hospitals’ adoption of mHealth.

#### Environment

Government policy and external pressure have been shown to influence the organizational adoption of new technologies. Existing laws and regulations can critically impact the adoption of new technologies [29,37]. In the health care context, compliance with legislation and standards has been found to be critical in the adoption of RFID patient tracking [42]. Government regulation has been advocated to play a more important role in the Chinese context compared with other developed economies [49]. As hospitals in China are state-owned, government policy can encourage or discourage hospitals from adopting mHealth. On this basis, we proposed the following:

Hypothesis 9: Government policy will positively influence hospitals’ adoption of mHealth.

External pressure has been found to be one of the most significant determinants of organizational adoption of new technologies [51]. Health care organizations are under constant pressure to adopt and implement new technologies to become more efficient [53]. To coordinate care and steer patients away from emergency departments, hospitals are under pressure to adopt HIE [41]. Thus, we suggested the following:

Hypothesis 10: External pressure will positively influence hospitals’ adoption of mHealth.

## Methods

### Data Collection

An interviewer-administered survey was conducted to test the research model empirically. The questionnaire was piloted and refined through rigorous pretesting. In this phase, 8 public hospital managers were invited to participate in this study and comment on instrument clarity and question wording. As a result, construct measures were revised. In addition, system reliability [54] was emphasized by hospital directors as a missing variable that should be included in the research instrument. Here, we revised the research instrument to include system reliability as a further measure of the technology context. A company would choose not to adopt cloud computing because of the increased business risk associated with the uncertainty of service availability and reliability, especially if there are unexpected downtimes and disruptions [55]. People often would not prefer to use new technology because of concerns about the reliability and stability of the system [55]. System reliability is key when providing uninterrupted services as shown in a study by Pagani [56]. Thus, we expect system reliability to influence hospitals’ adoption of mHealth significantly.

A convenient snowballing approach was used in this study. Due to the difficulty in obtaining data from public hospitals in China, the authors focused on collecting data from 2 regions, namely Shanghai and Gansu. A total of 91 questionnaires were obtained, but 4 questionnaires were discarded because of missing data. As a result, 87 responses were included in the analysis. Respondents, from hospitals that are not willing to adopt mHealth, were classified as nonadopters, whereas respondents from hospitals that are willing to adopt mHealth in the next 3 years were classified as adopters. Table 3 shows the characteristics of the responding hospitals in terms of location, hospital level, hospital beds, and respondents’ positions. Hospitals in China are classified into 3 major tiers, with 3 subtiers within each major tier [29,57]. Level 3 hospitals provide specialized medical services in several departments, with level 3A hospitals being the most advanced. These hospitals have a minimum capacity of 500 beds. Level 2 hospitals are regional hospitals with 100 to 499 beds providing cross-community medical services. These are smaller in size and less advanced than those in level 3 hospitals. Level 1 hospitals provide basic health care facilities with a capacity of 20 to 99 beds.

The questionnaire was translated into Chinese official language (standard Mandarin) following the conventional forward-then-back-translation approach. This has taken into account local culture and dialect considerations when establishing conceptual equivalence between English and Chinese versions of the instrument [58].

**Table 3:** Hospitals’ and respondents’ characteristics.

Demographics			Adopters (n=50), n (%)	Nonadopters (n=37), n (%)
**Location**				
	Shanghai		37 (74)	9 (24)
	Gansu		13 (26)	28 (76)
**Public hospital level**				
	**<Level 3**		35 (70)	7 (19)
		Level 3A	29 (58)	5 (14)
		Level 3B	6 (12)	2 (5)
	**>Level 3**		15 (30)	30 (81)
		Level 2	12 (24)	24 (65)
		Level 1	3 (6)	6 (16)
**Hospitals beds**				
	500+		35 (70)	7 (19)
	100-499		12 (24)	24 (65)
	20-99		3 (6)	6 (16)
**Respondents’ job titles**				
	Directors of laboratory services		37 (74)	10 (27)
	Directors of IT^a^ department		6 (12)	9 (24)
	Directors of other departments		7 (14)	18 (49)

^a^IT: information technology.

### Measures

Measurement items were developed based on a comprehensive review of the literature and modified to suit the mHealth context in China. All measurement items are listed in Multimedia Appendix 1. The items for perceived usefulness, perceived ease of use, and system compatibility were adopted from Moore and Benbasat [59]. The measure for system security was developed from Kim et al [60], the measure for IT infrastructure was developed from Bhattacherjee and Hikmet [61], and the measure for system reliability was adapted from Goodhue and Thompson [54]. The items for TMS were adapted from Yap et al [62], and the items for organizational readiness were adapted from Grandon and Pearson [63]. The items for government policy were adapted from Chau and Tam [32], and the items for external pressure were adopted from Premkumar and Roberts [64]. A 5-point Likert scale ranging from *strongly disagree* to *strongly agree* was used for all measurement items with the exception of hospital size, which was classified into 3 major tiers [29,57].

## Results

### Validity and Reliability

The validity of construct measures was assessed using principal component analysis with orthogonal factor rotation. All factor loadings were above 0.5 [65]. Reliability was assessed using Cronbach α. All α coefficients exceeded .7 [65]. As shown in Table 4, factor analysis and α coefficient results indicate adequate validity and reliability of the measures.

The correlation matrix was examined for multicollinearity problems. Table 5 shows that none of the squared correlation coefficients are above the 0.9 level [65]. Table 6 shows that the variance inflation factor values are not greater than the cutoff value of 10 [65], indicating that multicollinearity is not a problem for this study.

**Table 4:** Factor analysis and reliability assessment.

Constructs and items	PU^a^	PE^b^	SC^c^	SS^d^	ITI^e^	SR^f^	TMS^g^	OR^h^	GP^i^	EP^j^
PU1	.603	—^k^	—	—	—	—	—	—	—	—
PU2	.825	—	—	—	—	—	—	—	—	—
PU3	.727	—	—	—	—	—	—	—	—	—
PU4	.878	—	—	—	—	—	—	—	—	—
PU5	.584	—	—	—	—	—	—	—	—	—
PU6	.821	—	—	—	—	—	—	—	—	—
PE1	—	.621	—	—	—	—	—	—	—	—
PE2	—	.820	—	—	—	—	—	—	—	—
PE3	—	.773	—	—	—	—	—	—	—	—
PE4	—	.631	—	—	—	—	—	—	—	—
PE5	—	.739	—	—	—	—	—	—	—	—
PE6	—	.707	—	—	—	—	—	—	—	—
SC1	—	—	.694	—	—	—	—	—	—	—
SC2	—	—	.836	—	—	—	—	—	—	—
SC3	—	—	.663	—	—	—	—	—	—	—
SS1	—	—	—	.930	—	—	—	—	—	—
SS2	—	—	—	.896	—	—	—	—	—	—
SS3	—	—	—	.832	—	—	—	—	—	—
ITI1	—	—	—	—	.688	—	—	—	—	—
ITI2	—	—	—	—	.859	—	—	—	—	—
ITI3	—	—	—	—	.721	—	—	—	—	—
SR1	—	—	—	—	—	.821	—	—	—	—
SR2	—	—	—	—	—	.956	—	—	—	—
SR3	—	—	—	—	—	.772	—	—	—	—
TMS1	—	—	—	—	—	—	.719	—	—	—
TMS2	—	—	—	—	—	—	.819	—	—	—
TMS3	—	—	—	—	—	—	.897	—	—	—
OR1	—	—	—	—	—	—	—	.877	—	—
OR2	—	—	—	—	—	—	—	.859	—	—
OR3	—	—	—	—	—	—	—	.628	—	—
GP1	—	—	—	—	—	—	—	—	.873	—
GP2	—	—	—	—	—	—	—	—	.880	—
EP1	—	—	—	—	—	—	—	—	—	.702
EP2	—	—	—	—	—	—	—	—	—	.823
EP3	—	—	—	—	—	—	—	—	—	.895
Eigenvalue	2.748	4.330	4.039	2.653	2.367	2.686	2.250	2.152	1.711	1.355
Variance	7.634	12.028	11.221	7.369	6.576	7.460	6.251	5.978	4.752	3.764
Cronbach α	.778	.778	.786	.797	.794	.778	.764	.778	.790	.777

^a^PU: perceived usefulness.

^b^PE: perceived ease of use.

^c^SC: system compatibility.

^d^SS: system security.

^e^ITI: information technology infrastructure.

^f^SR: system reliability.

^g^TMS: top management support.

^h^OR: organizational readiness.

^i^GP: government policy.

^j^EP: external pressure.

^k^Constructs and items.

**Table 5:** Correlations between independent variables.

Constructs	PU^a^	PE^b^	SC^c^	SS^d^	ITI^e^	SR^f^	TMS^g^	OR^h^	HS^i^	GP^j^	EP^k^
PU	1.000	N/A^l^	N/A	N/A	N/A	N/A	N/A	N/A	N/A	N/A	N/A
PE	−0.277	1.000	N/A	N/A	N/A	N/A	N/A	N/A	N/A	N/A	N/A
SC	−0.247	0.394	1.000	N/A	N/A	N/A	N/A	N/A	N/A	N/A	N/A
SS	0.005	0.512	0.278	1.000	N/A	N/A	N/A	N/A	N/A	N/A	N/A
ITI	0.054	−0.440	−0.366	−0.368	1.000	N/A	N/A	N/A	N/A	N/A	N/A
SR	−0.054	−0.734	−0.481	−0.497	0.507	1.000	N/A	N/A	N/A	N/A	N/A
TMS	−0.235	0.659	0.489	0.454	−0.594	−0.694	1.000	N/A	N/A	N/A	N/A
OR	.048	0.456	0.440	0.476	−0.496	−0.612	0.516	1.000	N/A	N/A	N/A
HS	−0.122	0.531	0.384	0.471	−0.202	−0.554	0.567	0.269	1.000	N/A	N/A
GP	0.250	−0.786	−0.579	−0.575	0.470	0.708	−0.824	−0.634	−0.562	1.000	N/A
EP	−0.098	0.469	0.332	0.399	−0.207	−0.496	0.512	0.187	0.575	−0.643	1.000

^a^PU: perceived usefulness.

^b^PE: perceived ease of use.

^c^SC: system compatibility.

^d^SS: system security.

^e^ITI: information technology infrastructure.

^f^SR: system reliability.

^g^TMS: top management support.

^h^OR: organizational readiness.

^i^HS: hospital size.

^j^GP: government policy.

^k^EP: external pressure.

^l^N/A: not applicable.

**Table 6:** Collinearity statistics.

Constructs	Tolerance	Variance inflation factor
Perceived usefulness	0.638	1.567
Perceived ease of use	0.528	1.896
System compatibility	0.735	1.360
System security	0.841	1.188
Information technology infrastructure	0.742	1.347
System reliability	0.567	1.764
Top management support	0.475	2.106
Organizational readiness	0.564	1.773
Hospital size	0.742	1.347
Government policy	0.564	1.773
External pressure	0.543	1.840

### Model Testing

Logistic regression was used as the dependent variable was dichotomous (nonadopters vs adopters). This technique has been utilized in previous studies on the organizational adoption of technologies such as mobile reservation systems [38], electronic supply chain management [30], and enterprise systems [35].

Table 7 shows the results of the logistic regression analysis. The chi-square test was significant (omnibus X^2^_11_=70.4; *P*<.001), and 2 pseudo R^2^ values (Cox and Snell R^2^=0.55; Nagelkerke R^2^=0.74) were satisfactory. Moreover, the research model correctly predicted 81% (30/37) of the nonadopters and 88% (44/50) of the adopters with an overall predictive accuracy of 85% (Table 8). Overall, the research model exhibits an acceptable fit with the data.

**Table 7:** Results of the logistic regression.

Constructs^a^	β coefficient	Wald statistic	*P* value
Perceived usefulness	−.096	0.424	.51
Perceived ease of use	.692^b^	9.406	.002
System compatibility	.561	3.083	.07
System security	.473^c^	3.828	.05
Information technology infrastructure	−.574^c^	4.784	.02
System reliability	−1.291^c^	6.123	.01
Top management support	1.466^b^	9.614	.002
Organizational readiness	.605	2.170	.14
Hospital size	1.069^b^	8.345	.004
Government policy	−2.010^c^	6.516	.04
External pressure	.703^b^	3.972	.005

^a^Goodness-of-fit: omnibus X^2^_11_=70.4; *P*<.001; −2 log likelihood value=118.658; Cox and Snell R^2^=0.55; Nagelkerke R^2^=0.74.

^b^*P*<.01.

^c^*P*<.05.

**Table 8:** Discriminating power.

Observed	Predicted		Percentage correct
	Nonadopters, n (%)	Adopters, n (%)	
Nonadopters	30 (81)	7 (19)	81
Adopters	6 (12)	44 (88)	88
Overall	N/A^a^	N/A	85

^a^N/A: not applicable.

As shown in Table 7, perceived ease of use (β=.692; *P*<.002), system security (β=.473; *P*<.05), TMS (β=1.466; *P*<.002), hospital size (β=1.069; *P*<.004), and external pressure (β=.703; *P*<.005) were significantly related to hospitals’ adoption of mHealth. IT infrastructure (β=−.574; *P*<.02), system reliability (β=−1.291; *P*<.01), and government policy (β=−2.010; *P*<.04) were significant but negatively related to hospitals’ adoption of mHealth. Thus, hypotheses 2, 4, 6, 8, and 10 are supported. However, perceived usefulness, system compatibility, and organizational readiness did not exhibit a significant relationship with hospitals’ adoption of mHealth. Thus, hypotheses 1, 3, and 7 are not supported. These findings are summarized in Table 9.

**Table 9:** Summary of hypotheses support.

Predictors	Hospital mobile health adoption
Perceived usefulness	Reject
Perceived ease of use	Accept
System compatibility	Reject
System security	Accept
Information technology infrastructure	Reject (significant but negative)
System reliability	Reject (significant but negative)
Top management support	Accept
Organizational readiness	Reject
Hospital size	Accept
Government policy	Reject (significant but negative)
External pressure	Accept

## Discussion

### Principal Findings

This study explores the determinants of hospitals’ adoption of mHealth using the TOE framework. The technological determinants of mHealth adoption by hospitals were examined. Although perceived ease of use and system security are facilitators, IT infrastructure and system reliability are inhibitors of mHealth adoption by hospitals. Perceived ease of use has been found to be a significant determinant of mHealth adoption not only among diabetic patients [66] but also among health care professionals [67]. In addition, security and privacy protection has been found to influence hospital adoption of mHealth [66] and RFID patient tracking [42]. Lack of security was found to be a barrier to telemedicine adoption by physicians [68]. Unexpectedly, IT infrastructure and system reliability were significant but negatively related to hospitals’ adoption of mHealth. These results differ from those obtained by Vest [41], who noted that hospitals with low levels of IT infrastructure readiness have lower odds of HIE adoption. They also differ from the results obtained by Shareef et al [66], who found that perceived reliability is positively associated with mHealth adoption. A possible explanation for the negative relationships is the lack of a comprehensive strategy to invest, implement, and use mHealth. Evidence indicates that even among hospitals with established strategies to adopt mHealth solutions, only a few attempt to integrate and align these mHealth solutions with their existing IT systems [69].

Surprisingly, perceived usefulness does not exhibit a positive effect on hospitals’ adoption of mHealth. The reason for this insignificant result could be because of the lack of awareness of the benefits of adopting mHealth solutions. This finding is similar to that of Wang et al [45] study of RFID adoption by manufacturing firms and the study [38] of adoption of mobile reservation systems by hotels. In addition, system compatibility does not exhibit a positive effect on hospitals’ adoption of mHealth. Here, hospital managers seem to underestimate the significance of system compatibility and the extent to which mHealth is perceived to be consistent with their needs, values, and experiences. A possible explanation for this insignificant result is that new mHealth technologies can be easily integrated with existing systems. This finding is similar to that of both study of enterprise systems adoption by SMEs by Ramdani et al [35] and the study of cloud computing adoption in manufacturing and services firms by Oliveira et al [40].

Organizational determinants of mHealth adoption by hospitals were investigated. As expected, TMS and hospital size are facilitators of mHealth adoption by hospitals. These findings are consistent with Cao et al [42], who found management support to be key to the success of RFID application in hospitals, and Hung et al [23], who found hospital size to be a critical factor in CRM adoption by hospitals. Surprisingly, organizational readiness does not exhibit a significant effect on hospitals’ adoption of mHealth. Although lack of financing has been found to be a barrier to the adoption of 3 health information technologies, including electronic health record functionalities, electronic-prescriptions, and telemedicine [68], the reason behind the insignificance of organizational readiness is that mHealth technologies do not require a substantial upfront investment. The cost associated with mHealth tends to be much lower than that of traditional medical services [12].

The environmental determinants of mHealth adoption by hospitals were investigated. Although government policy is an inhibitor, external pressure is a facilitator of mHealth adoption by hospitals. Government policy is significant but negatively associated with hospitals’ adoption of mHealth. The lack of an enabling health care policy has been suggested as a barrier to mHealth adoption [70]. Furthermore, the current highly regulated environment in hospitals in China could hinder the adoption of new technologies. As expected, external pressure is positively associated with hospital adoption of mHealth. This result is supported by Cao et al [42] study of RFID adoption in the health care sector, where external pressure was found to be one of the key dimensions of the environmental context. Moreover, competition between health care organizations has been found to be a key determinant of HIE adoption [41].

### Study Implications and Limitations

The results of this study provide practical implications for mHealth suppliers and policymakers. First, both perceived ease of use and system security in the technological context have a significant effect on hospitals’ adoption of mHealth. To facilitate hospital adoption, mHealth developers and suppliers need to ensure that the adoption process is relatively simple, and the system is highly secure. Second, both TMS and hospital size in the organizational context have a significant positive effect on hospitals’ adoption of mHealth. To get hospitals to adopt mHealth, suppliers need to direct their advertising and promotions toward senior executives who make the final decision to adopt. In addition, mHealth suppliers may need to target larger hospitals because they are likely to invest in such systems. Finally, government policy and external pressure in the environmental context have a significant effect on hospitals’ adoption of mHealth. Although external pressure facilitates the adoption of mHealth, existing government policies must be revised to encourage the adoption of new technologies in the health care sector.

Several potential limitations must be considered when interpreting the results of this study. First, this study focuses only on hospitals’ adoption of mHealth. The impact of mHealth on hospital performance should be examined to gain a holistic understanding. Second, a set of technological, organizational, and environmental predictors were examined. Future studies may examine whether other predictors may influence hospitals’ adoption of mHealth. Third, the collected data are cross-sectional and posited causal relationships could only be inferred. Future studies could collect longitudinal data to determine causal links more explicitly. Fourth, the statistical technique employed (ie, logistic regression) only analyses the relationships between hospitals' adoption of mHealth and their predictors and does not analyze the relationships between the predictors. Future studies could use other statistical techniques to examine the relationships between the predictors and elaborate on the findings of this study. Another important limitation lies in the sample size and type of hospitals studied. Although our sample size was adequate for this study, the findings might vary with larger samples. In addition, because private hospitals are developing rapidly in China, it will be worth examining how our findings compare with private hospitals’ adoption of mHealth. Finally, our data are largely dominated by hospitals from 2 regions in China: Shanghai and Gansu. We have overlooked the potential regional differences in our study. Thus, the research model should be tested further using larger samples from other regions or even from other counties to make cross-region or cross-country comparisons.

### Conclusions

This study contributes to the literature on organizational mHealth adoption by examining the determinants of mHealth adoption by hospitals. The results indicate that significant predictors of hospitals' adoption of mHealth include perceived ease of use, system security, IT infrastructure, system reliability, TMS, hospital size, external pressure, and government policy. However, perceived usefulness and system compatibility in the technological context and organizational readiness in the organizational context are not significant predictors.

The contributions of this study to research on organizational mHealth adoption are 3-fold. First, previous studies focused on the adoption of mHealth at an individual level, including health care professionals and patients. This study adds important insights to the literature by focusing on the organizational (ie, hospital) adoption of mHealth. Second, limited knowledge exists on the adoption of technology in Chinese health care organizations. This study contributes to the literature by highlighting the context-specific determinants of mHealth adoption. Third, studies of adoption of technology in health care organizations mainly use versions of the unified theory of acceptance and use of technology framework. This study uses the TOE framework to contribute to the adoption of technology in the health care literature by identifying the predictors that influence hospitals to adopt mHealth.

## Appendix

Multimedia Appendix 1 Questionnaire: Measurement of constructs.

## Abbreviations

CRM: customer relationship management
e-business: electronic business
HIE: health information exchange
IT: information technology
mHealth: mobile health
RFID: radio-frequency identification
SME: small-to-medium–sized enterprise
TMS: top management support
TOE: technology-organization-environment
