# Supplementary material for: Exploring the Determinants of Mobile Health Adoption by Hospitals in China: Empirical Study
Source: JMIR Med Inform. 2020 Jul 14;8(7):e14795. doi: 10.2196/14795 (PMC7388041; doi:10.2196/14795)
Supplement: Multimedia Appendix 1 [file medinform_v8i7e14795_app1.docx]

## Multimedia Appendix 1

Questionnaire: Measurement of constructs.

*Perceived Usefulness*

PU1: Using mHealth can enable our healthcare professionals to complete patient care more quickly.

PU2: Using mHealth CANNOT improve our healthcare professionals’ patient care and management.

PU3: Using mHealth can increase our healthcare professionals’ productivity in patient care.

PU4: Using mHealth CANNOT enhance our healthcare professionals’ service effectiveness.

PU5: Using mHealth can make our healthcare professionals’ patient care and management easier.

PU6: Our healthcare professionals would find mHealth NOT useful for their patient care and management.

*Perceived Ease-of-Use*

PE1: Learning to use mHealth would NOT be easy for our healthcare professionals.

PE2: Our healthcare professionals would find it easy to get mHealth to do what they need to do in their patient care and management.

PE3: Our healthcare professionals’ interaction with mHealth would be clear and understandable.

PE4: I find mHealth INFLEXIBLE to interact with.

PE5: It is NOT easy for our healthcare professionals to become skilful in using mHealth.

PE6: Our healthcare professionals would find mHealth easy to use.

*System Compatibility*

SC1: The changes introduced by mHealth are consistent with my hospital’s existing beliefs/values.

SC2: mHealth is compatible with my hospital’s existing information infrastructure.

SC3: The changes introduced by mHealth are consistent with my hospital’s existing practice.

*System Security*

SS1: mHealth will not provide patients’ personal information to other entities without their consent.

SS2: mHealth will not cause the use of patients’ personal information for other purposes without their authorisation.

SS3: mHealth has mechanisms to ensure the safe transmission of patients’ information.

*IT Infrastructure*

ITI1: The level of IT provided at our hospital is adequate to perform our job.

ITI2: Our hospital is keeping up with advances in IT.

ITI3: Our hospital would provide additional IT products to improve the quality of our work.

*System Reliability*

SR1: Our healthcare professionals can count on the system to be "up" and available when they need it.

SR2: The mHealth system our healthcare professionals use are subject to unexpected or inconvenient down times which make it harder to do my work.

SR3: The mHealth system our healthcare professionals use are subject to frequent problems and crashes.

*Top Management Support*

TMS1: Our top management is likely to be interested in adopting mHealth in order to gain competitive advantage.

TMS2: Our top management is willing to take the risks involved in the adoption of mHealth.

TMS3: Our top management is likely to invest funds in mHealth.

*Organisational Readiness*

OR1: We have the resources necessary to adopt mHealth.

OR2: We have the knowledge necessary to use the system.

OR3: A specific person (or group) is available for assistance with system difficulties.

*Government Policy*

GP1: The legislative regulation pledges your hospital to use mHealth.

GP2: The compliance with the legislative regulations regarding mHealth is strictly enforced.

*External Pressure*

EP1: It is very important for our hospital to adopt mHealth to remain competitive.

EP2: Most hospitals use mHealth for daily jobs.

EP3: Currently, our hospital’s competitors are adopting mHealth.
